# Meeting Report from the Genomic Standards Consortium (GSC) Workshops 6 and 7

**DOI:** 10.4056/sigs.25165

**Published:** 2009-07-20

**Authors:** Dawn Field, Peter Sterk, Nikos Kyrpides, Renzo Kottmann, Frank Oliver Glöckner, Lynette Hirschman, George M. Garrity, John Wooley, Paul Gilna

**Affiliations:** 1 NERC Center for Ecology and Hydrology, Oxford, United Kingdom; 2 The Pathogen Sequencing Unit, The Wellcome Trust Sanger Institute, Wellcome Trust Genome Campus, Hinxton, Cambridge, UK; 3 Genome Biology Program, DOE Joint Genome Institute, Walnut Creek, California, USA; 4 Microbial Genomics Group, Max Planck Institute for Marine Microbiology & Jacobs University Bremen, Bremen, Germany; 5 Information Technology Center, The MITRE Corporation, Bedford, Massachusetts, USA; 6 Department of Microbiology and Molecular Genetics, Michigan State University, East Lansing, Michigan, USA; 7 University of California San Diego, La Jolla California

## Abstract

This report summarizes the proceedings of the 6th and 7th workshops of the Genomic Standards Consortium (GSC), held back-to-back in 2008. GSC 6 focused on furthering the activities of GSC working groups, GSC 7 focused on outreach to the wider community. GSC 6 was held October 10-14, 2008 at the European Bioinformatics Institute, Cambridge, United Kingdom and included a two-day workshop focused on the refinement of the Genomic Contextual Data Markup Language (GCDML). GSC 7 was held as the opening day of the International Congress on Metagenomics 2008 in San Diego California. Major achievements of these combined meetings included an agreement from the International Nucleotide Sequence Database Consortium (INSDC) to create a “MIGS” keyword for capturing ”Minimum Information about a Genome Sequence” compliant information within INSDC (DDBJ/EMBL /Genbank) records, launch of GCDML 1.0, MIGS compliance of the first set of “*Genomic Encyclopedia of Bacteria and Archaea*” project genomes, approval of a proposal to extend MIGS to 16S rRNA sequences within a “Minimum Information about an Environmental Sequence”, finalization of plans for the GSC eJournal, “*Standards in Genomic Sciences*” (SIGS), and the formation of a GSC Board. Subsequently, the GSC has been awarded a Research Co-ordination Network (RCN4GSC) grant from the National Science Foundation, held the first SIGS workshop and launched the journal. The GSC will also be hosting outreach workshops at both ISMB 2009 and PSB 2010 focused on “Metagenomics, Metadata and MetaAnalysis” (M^3^). Further information about the GSC and its range of activities can be found at http://gensc.org, including videos of all the presentations at GSC 7.

## Introduction

The Genomic Standards Consortium (GSC) is an initiative working towards richer descriptions of our collection of genomes and metagenomes through the development of standards and tools for supporting compliance and exchange of contextual information [1]. Established in September 2005, this international community includes representatives from the International Nucleotide Sequence Database Collaboration (INSDC), major genome sequencing centers, bioinformatics centers and a range of research institutions.

The rapid pace of genomic and metagenomic sequencing projects [2], which now include studies of microbiomes, will only increase as the use of ultra-high-throughput sequencing methods becomes more common place. Therefore, the role of standards becomes even more vital to scientific progress and data sharing. It is clear that we need new standards to capture additional contextual data as well as tools to support its use in downstream computational analyses. The GSC aims to hold workshops designed to allow the community to advance identified GSC projects and propose new ones. Face-to-face workshops also help to grow GSC membership and broaden linkages between the GSC and related projects within the wider scientific commons. A brief overview of the highlights of GSC 6 and 7 is given below.

## GSC 6: Implementation of MIGS

### GCDML Workshop

The GSC 6 workshop opened with a two day GCDML workshop at which GCDML version v1.0 was presented and discussed in depth by about 20 attendees. The workshop, led by Renzo Kottmann (MPI-Bremen), was designed to inform developers within the GSC of the design and construction of GCDML with the goal of accelerating its adoption and extending its content. The workshop included sessions on how to create and edit genome reports using GCDML markup in an XML Editor. Examples of 30 marine phage genomes marked up in GCDML by Melissa Beth Duhaime (MPI-Bremen) were used to illustrate the creation and maintenance of GCDML instances.

Discussions of GCDML and the vision of MIGS/MIMS compliance by the community in the near future led to renewed interest in building the GSC Genome Catalogue [1]. A comprehensive catalogue could act as a central hub of information accessible by web services and linked to core databases maintained by participating GSC organizations, many of which already collect, or soon will collect MIGS/MIMS metadata. Participants agreed to create a community-led requirements document describing an ideal future solution. It was agreed that, at a minimum, the Genome Catalogue should be:

- Funded (a long-term endeavor that can not be done on a voluntary basis)
- Based on GCDML
- Underpinned by a rich, user-friendly tool kit
- Shared by the GSC
- Designed to give credit to all contributors
- Expressed in XML using GCDML syntax
- Web services based (supporting the automated exchange of content)
- Serve as the international GCAT identifier authority (for Genome Catalogue entries)
- Comprehensive (containing reports for all taxa and metagenomes)
- Ontology-supportive
- Able to maintain all versions of GCDML schemas used to curate metadata

The workshop closed with agreement that the focus for 2009 should be curation of MIGS/MIMS metadata for key sets of genomes. Peter Sterk is now leading this effort.

### GSC 6: Main Meeting

The GCDML workshop provided an excellent foundation for the main meeting, which was structured into six sessions held over three days. The first day of this meeting was spent reviewing ongoing GSC activities and developments since the GSC 5 workshop [1]. The “*Minimum Information about a (Meta) Genome Seque*nce” (MIGS) appeared in print in June 2008 [2]. A special issue of *OMICS* was published as a result of GSC 5 [3] containing roadmap papers on core GSC activities including: GCDML [4], the Genomic Rosetta Stone mapping of genomic identifiers [5], Habitat-Lite [6]and the GSC eJournal [7,8]. These roadmaps place the GSC firmly in Phase II, which will center on implementation in aid of the adoption of the MIGS specification [2] now that the GSC has built it and presented to the community in Phase I of the evolution of the GSC. As done previously, the final day was dedicated to the development of the next leg of the GSC strategy.

The full agenda of the workshop, which was attended by more than 40 invited participants, is available on line and included a line-up of excellent speakers and talks. Only the major highlights of the meeting are covered in this brief overview and include:

- **INSDC agreement** (Guy Cochrane, EMBL, Ilene Mizrachi and Scott Federhan, NCBI) to take forward a proposal to allow the GSC create a reserved keyword “MIGS” for inclusion in INSDC submission files following the precedent set previously by the CBoL (the Consortium for Barcodes of Life) This proposal was approved at the INSDC annual meeting in May 2009
- **Discussion of the MIGS checklist** (Nikos Kyrpides, DOE Joint Genome Institute) and finalization of MIGS information for the first 60 genomes to be published by the “*Genomic Ency-clopedia of Bacteria and Archaea*” project
- **Approval of a proposal** (Frank Oliver Glöckner, MPI-Bremen) to extend MIGS/MIMS to ribosomal RNA that will be defined by a newly developed “*Minimum Information about an Environmental Sequence*”. This will be a major focus of GSC 8.
- **Agreement to name the GSC eJournal***Standards in Genomic Sciences* (SIGS) and recruitment of editors (George Garrity, Michigan State University)

Key actions and designated project leads agreed to by the group included:

- **Development of MIGS/MIMS content** in coming months and publication of requirements for a GSC Genome Catalogue (Peter Sterk).
- Incorporation of a MIGS keyword into INSDC records (Peter Sterk, Guy Cochrane and Nikos Kyrpides)
- **Compilation of MIGS 2.1,** with the view that the checklist would be maintained as a function of SIGS (Peter Sterk).
- **Improvement of the GSC wiki content** (Dawn Field, Peter Sterk and Renzo Kottmann).
- **Initiation of publication of SIGS,** including recruitment of authors, editors and reviewers (George Garrity)
- **Further engagement of the broader community** through a Special Interest Group workshop at the Intelligent Systems in Molecular Biology (ISMB 2009) conference (Iddo Friedberg)

### GSC 7: Community engagement at the Metagenomics ‘08

This one-day ‘community outreach’ event was held on the opening day of the much larger Metagenomics ‘08 conference. Attendees included more than 100 participants who were not yet members of the GSC. This was an excellent forum for the GSC to present its full range of ideas and activities to the wider community, in a persistent manner since the presentations were recorded as videos distributed through both SciVee and the GSC website. In addition to the 17 presentations by GSC members, an open session was held the following day to discuss GSC projects, opportunities and business. This second chance to meet face-to-face in 2008 led to the formulation of a successful M3 proposal for the ISMB/ECCB meeting in Stockholm in June 2009, which was led by Dawn Field (NERC Centre for Ecology and Hydrology) and the establishment of a GSC Board comprised of long-standing GSC members.

### Post meeting activities: The future of the GSC

Since these workshops, the GSC has been awarded a Research Co-ordination Network grant, led by John Wooley (UCSD), Dawn Field, and Frank Oliver Glöckner, from the National Science Foundation (NSF). These funds will allow the GSC to continue holding face-to-face meetings (large and small) and to support the exchange of bioinformaticians working on GSC projects between labs. George Garrity hosted the first SIGS workshop at Michigan State University (March 2009) (see this issue) to address technical and organizational issues prior to the launch of the first issue of the journal. In addition to the successful ISMB 2009 proposal, the “M3” workshop concept was submitted to and accepted by PSB 2010 by Iddo Friedberb (UCSD and Dawn Field. Peter Sterk completed the curation of MIGS metadata in GCDML format for all published Sanger genomes. These have been submitted to the European Nucleotide Archive (ENA) with the help of Guy Cochrane. Nikos Krypides, Dawn Field, Peter Sterk and John Wooley, with the support of the newly established GSC Board, have agreed to organize GSC 8 at the DOE Joint Genome Institute in September 2009. With the help of Eugene Kolker and other members of the GSC Board, the GSC has become a legally chartered nonprofit organization, head-quartered in Seattle, Washington.
